# Effects of Hyperbaric Oxygen Preconditioning on Doxorubicin Cardiorespiratory Toxicity

**DOI:** 10.3390/antiox11102073

**Published:** 2022-10-20

**Authors:** Vivian Doerr, Ryan N. Montalvo, Branden L. Nguyen, Franccesco P. Boeno, Michael D. Sunshine, Victoria E. Bindi, David D. Fuller, Ashley J. Smuder

**Affiliations:** 1 Department of Applied Physiology and Kinesiology, University of Florida, Gainesville, FL 32611, USA; 2 Breathing Research and Therapeutics Center, University of Florida, Gainesville, FL 32610, USA; 3 Department of Physical Therapy, University of Florida, Gainesville, FL 32611, USA; 4 McKnight Brain Institute, University of Florida, Gainesville, FL 32610, USA

**Keywords:** doxorubicin, hyperbaric oxygen therapy (HBO), cardiorespiratory, antioxidants

## Abstract

Cardiorespiratory dysfunction resulting from doxorubicin (DOX) chemotherapy treatment is a debilitating condition affecting cancer patient outcomes and quality of life. DOX treatment promotes cardiac and respiratory muscle pathology due to enhanced reactive oxygen species (ROS) production, mitochondrial dysfunction and impaired muscle contractility. In contrast, hyperbaric oxygen (HBO) therapy is considered a controlled oxidative stress that can evoke a substantial and sustained increase in muscle antioxidant expression. This HBO-induced increase in antioxidant capacity has the potential to improve cardiac and respiratory (i.e., diaphragm) muscle redox balance, preserving mitochondrial function and preventing muscle dysfunction. Therefore, we determined whether HBO therapy prior to DOX treatment is sufficient to enhance muscle antioxidant expression and preserve muscle redox balance and cardiorespiratory muscle function. To test this, adult female Sprague Dawley rats received HBO therapy (2 or 3 atmospheres absolute (ATA), 100% O_2_, 1 h/day) for 5 consecutive days prior to acute DOX treatment (20 mg/kg i.p.). Our data demonstrate that 3 ATA HBO elicits a greater antioxidant response compared to 2 ATA HBO. However, these effects did not correspond with beneficial adaptations to cardiac systolic and diastolic function or diaphragm muscle force production in DOX treated rats. These findings suggest that modulating muscle antioxidant expression with HBO therapy is not sufficient to prevent DOX-induced cardiorespiratory dysfunction.

## 1. Introduction

Hyperbaric oxygen (HBO) is a therapy used in clinical practice to treat ischemic diseases, decompression sickness and to promote wound healing [1,2,3]. Exposure to 100% oxygen at increased atmospheric pressure above 1 absolute atmosphere (ATA) enhances oxygen transport capacity of the blood, increasing oxygen delivery throughout the body [4]. Repeated daily bouts of HBO have been shown to induce physiological adaptations similar to exercise training, including improved cardiorespiratory function, fatigue resistance, increased antioxidant capacity and enhanced mitochondrial function [5,6,7,8,9,10]. Therefore, HBO treatment may have therapeutic potential to improve muscle function and quality of life in patients suffering from cardiorespiratory dysfunction.

Doxorubicin (DOX) is a chemotherapeutic agent used in the treatment of a variety of cancers. While DOX is highly effective at reducing tumor burden, its use is limited by off-target accumulation in muscle tissue and the subsequent development of cardiac dysfunction, dyspnea, exercise intolerance and fatigue [11,12]. Within muscle tissue, DOX primarily localizes to mitochondria, where redox cycling on complex I results in the production of superoxide anions and reactive oxygen species (ROS) [11]. In addition, DOX can induce mitochondrial permeability transition, inhibit oxidative phosphorylation and promote mitochondria-initiated cell death [13]. These effects of DOX on muscle mitochondrial function are key to triggering downstream events, ultimately causing reduced quality of life and increased mortality of cancer patients and survivors due to cardiorespiratory complications [13,14]. Evidence that HBO therapy can stimulate increased antioxidant expression, oxidative phosphorylation and improve cardiorespiratory function [15,16] supports the hypothesis that HBO preconditioning may be an effective therapy to prevent DOX-induced cardiorespiratory muscle dysfunction.

The clinical benefits of HBO preconditioning have been demonstrated in human patients with coronary heart disease prior to on-pump cardiac surgery resulting in improved ventricular ejection and reduced myocardial injury [17]. Furthermore, when used as a preventative method, HBO has been shown to improve ischemic tolerance of the brain, spinal cord, liver, heart, and skeletal muscle through increases in antioxidant enzymes and anti-inflammatory proteins [6,18,19,20,21]. However, evidence on the effects of HBO given in combination with DOX treatment is mixed and poorly understood [22,23,24,25,26,27]. This study adds to the limited understanding regarding the safety and efficacy of HBO as a therapy to combat DOX myotoxicity.

## 2. Materials and Methods

### 2.1. Animals

4-month-old female Sprague Dawley rats (Charles River Laboratories) were used in these experiments. Female rats were chosen due to the prevalence of DOX in the treatment of breast and gynecological cancers [28,29]. Experimental groups (n = 6/group) include: (1) 5 days room air exposure, saline treatment (CON); (2) 5 days room air exposure, DOX treatment (DOX); (3) 5 days 2 ATA HBO exposure, DOX treatment (2ATA-DOX); and (4) 5 days 3 ATA HBO exposure, DOX treatment (3ATA-DOX). Animals were housed at the University of Florida Animal Care Services Center and maintained under a 12:12 h light/dark cycle. Food and water were provided ad libitum throughout the experimental period. The Institutional Animal Care and Use Committee of the University of Florida approved these experiments (protocol no. 201910975).

### 2.2. Hyperbaric Oxygen Treatment

Animals were exposed to HBO for one hour per day for 5 days. A custom 32 L chamber was flushed with 100% O_2_ (i.e., 1 ATA O_2_) and then pressurized to either 2 or 3 ATA (~5 psi/min). These pressures were chosen based on reports indicating potential dose dependency for HBO pressure and that 3 ATA is a safe maximal pressure for HBO therapy [8,30]. During HBO exposure, the chamber was continuously flushed with O_2_ (4 L/min) to prevent CO_2_ buildup. Animals breathing room air were exposed to the non-pressurized chamber an equal period of time as the HBO treated rats. Twenty-four hours following the last HBO or room air exposure, animals received a single injection of DOX or saline via intraperitoneal (i.p.) injection.

### 2.3. DOX Treatment

Animals received either a 20 mg/kg i.p. dose of DOX (McKesson, Irving, TX, USA) or an equal volume of saline. Forty-eight hours following DOX or saline treatment, cardiac ultrasound was performed immediately prior to sacrifice. Ex vivo diaphragm force production and mitochondrial function (heart and diaphragm) were measured, a strip of diaphragm muscle was stored in OCT for cross-sectional area (CSA) analysis, and the remaining diaphragm, heart and plasma were stored at −80 °C for subsequent analysis.

### 2.4. Echocardiography

Transthoracic echocardiography was performed and analyzed by a single blinded author as previously described [31] utilizing a LogiQe NextGen ultrasound (SOUND Technologies, Carlsbad, CA, USA) and ImageJ (NIH, Bethesda, MD, USA). In brief, animals were anesthetized with inhaled isoflurane during which 2D ultrasound images and M-mode tracings of the left ventricle (LV) were obtained in the parasternal short-axis view at the level of the papillary muscles. Measurements were obtained over 10–15 cardiac cycles and averaged for each animal. Systolic function was evaluated by fractional shortening percentage. Myocardial performance index (MPI), measured via Doppler ultrasound, and posterior wall shortening velocity (PWSV) was utilized to measure combined systolic and diastolic function of the LV. Following echocardiography, animals were euthanized and tissues were collected for evaluation of outcome measures.

### 2.5. Plasma Collection and Analysis

Upon sacrifice, ~3 mL blood samples were collected via cardiac puncture from the left ventricle and placed in K_3_ EDTA blood collection tubes. All blood samples were centrifuged at 5000 rpm for 10 min at 4 °C. Plasma was collected and immediately stored at −80 °C. Plasma cytokine and chemokine concentrations were measured according to manufacturer’s instructions, using a Luminex Magpix^®^ multiplex analyzer (27 Plex MILLIPLEX MAP Rat Cytokine/Chemokine Magnetic Bead Panel, Millipore Sigma, Burlington, MA, USA). Twenty-five microliters of undiluted plasma samples were run in duplicate, and the assay was analyzed using Belysa™ Immunoassay Curve Fitting Software (Millipore Sigma).

A separate set of female Sprague Dawley control rats of similar age were used to collect pre- and post-HBO plasma samples (n = 7–9/group). A catheter was placed in the tail vein prior to HBO exposure to collect pre-HBO blood samples. Animals were placed inside the HBO chamber, which was flushed with 100% O_2_ and then pressurized to either 2 or 3 ATA. Immediately after the 1-h exposure, animals were removed from the chamber and blood was collected via the tail vein catheter. Plasma samples were prepared as described above and assayed via the 27 Plex MILLIPLEX MAP Rat Cytokine/Chemokine Magnetic Bead Panel.

### 2.6. Diaphragm Force Production and Fatigue

A muscle strip was immediately dissected upon sacrifice from the mid-costal region of the diaphragm, including the tendinous attachments at the central tendon and rib cage. The strip was suspended vertically with one end connected to an isotonic force transducer (Aurora Scientific, Aurora, ON, Canada) within a jacketed tissue bath at 25 °C. Diaphragm contractile properties were measured as previously described [32]. To measure the force-frequency response, each strip was stimulated supramaximally at optimal length with 120-V pulses at 15–160 Hz [33]. To measure the rate of muscle fatigue, the diaphragm strip was stimulated by unfused tetanic contractions using a stimulus train of 30 Hz every 2 s for 600 s with a train duration of 250 ms. Muscle fatigue is expressed as the time for diaphragm force production to reach 60% of its initial force.

### 2.7. Diaphragm Cross-Sectional Area Analysis

Diaphragm muscle cross-sections (10 μm) were cut using a cryostat (CM3050S Cryostat, Leica Biosystems, Wetzlar, Germany) and immunohistochemistry was performed for fiber CSA as described previously [34]. Sections were stained for α-laminin (L9393) (Millipore Sigma), myosin heavy chain type I (A4.840) (Developmental Studies Hybridoma Bank (DSHB), Iowa City, IA, USA) and myosin heavy chain type IIa (SC-71) (DSHB). Fluorescent images were acquired using a Revolve microscope (ECHO Laboratories, San Diego, CA, USA). CSA was analyzed with ImageJ software.

### 2.8. Permeabilized Muscle Fibers, Mitochondrial Respiration and ROS Emission

Mitochondrial respiration and ROS emission were measured in permeabilized heart and diaphragm fibers, using previously described techniques [35]. ~5–8 mg fiber bundles were teased apart in ice-cold Buffer X (in mM: 60 K-MES, 35 KCl, 7.23 K_2_EGTA, 2.77 CaK_2_EGTA, 20 imidazole, 0.5 dithiothreitol, 20 taurine, 5.7 ATP, 15 phosphocreatine, and 6.56 MgCl_2_; pH 7.1), permeabilized in Buffer X containing 50 μg/mL saponin, then washed for 15 min in Buffer Z (in mM: 110 K-MES, 35 KCl, 1 EGTA, 5 K_2_HPO_4_, 3 MgCl_2_, 0.05 glutamate, 0.02 malate, and 0.5 mg/mL bovine serum albumin (BSA) (pH 7.1). To measure respiration, permeabilized heart and diaphragm fiber bundles were placed in respiration chambers of an Oxygraph (Hansatech Instruments, King’s Lynn, UK) maintained at 37 °C, with 1 mL of Buffer Z containing 20 mM creatine to saturate creatine kinase. State 3 was initiated by adding 5 mM of pyruvate, 5 mM of malate and 0.25 mM ADP, followed by addition of 10 μg/mL oligomycin to inhibit ATP synthesis and determine state 4. The respiratory control ratio (RCR) was calculated by dividing state 3 by state 4 respiration. ROS emission in permeabilized heart and diaphragm muscle fibers was determined using Amplex Red (Molecular Probes, Eugene, OR, USA). Sample fluorescence was normalized to dry weight of the tissue.

### 2.9. Western Blot Analysis

Heart and diaphragm muscles were homogenized 1:10 (*w*/*v*) in 5 mM Tris/5 mM EDTA with a protease inhibitor cocktail (Millipore Sigma) and assayed as described [36]. The Bradford method (Millipore Sigma) was used to assess supernatant protein content. Proteins were separated via 4–20% precast gels (Bio-Rad Laboratories, Hercules, CA, USA) and transferred to nitrocellulose membranes, which were blocked with 5% non-fat milk and probed with primary antibodies diluted in Odyssey blocking buffer (LI-COR, Lincoln, NE, USA) for the following proteins of interest: glutathione peroxidase 1 (GPX1) #ab22604, catalase #ab16731 (Abcam, Cambridge, UK), peroxiredoxin III (PRXIII) #sc23973, superoxide dismutase 1 (SOD1) #sc11407, and superoxide dismutase 2 (SOD2) #sc30080 (Santa Cruz Biotechnology, Dallas, TX, USA). Protein abundance was normalized to glyceraldehyde 3-phosphate dehydrogenase (GAPDH) #sc47724 (Santa Cruz Biotechnology), which served as a loading control. Following overnight incubation with primary antibodies at 4 °C, membranes were washed with PBS with 0.05% Tween and then incubated with corresponding IRDye secondary antibodies (anti-mouse IgG 680RD #926-68070, anti-mouse IgG 800CW #926-32210, anti-rabbit IgG 680RD #926-68071, anti-rabbit IgG 800CW #926-32211, anti-goat IgG 800CW #926-32214; LI-COR). Membranes were imaged on an Odyssey CLx (LI-COR). Images were then analyzed using Image Studio^TM^ software version 5.2.5 (LI-COR).

### 2.10. Statistical Analysis

A Shapiro–Wilk test was used to test for normality. For repeated measures analysis, a two-way analysis of variance (ANOVA) was used to compare matched values. When significant, Sidak’s multiple comparisons test was used post hoc. For all other analyses, a one-way ANOVA with a Dunnett’s multiple comparisons test performed post hoc was used. Significance was established at *p* < 0.05. All data are presented as means ± standard error of mean (SEM).

## 3. Results

### 3.1. Plasma Cytokine/Chemokine Levels Pre- and Post-Acute HBO Exposure

**Figure 1:**
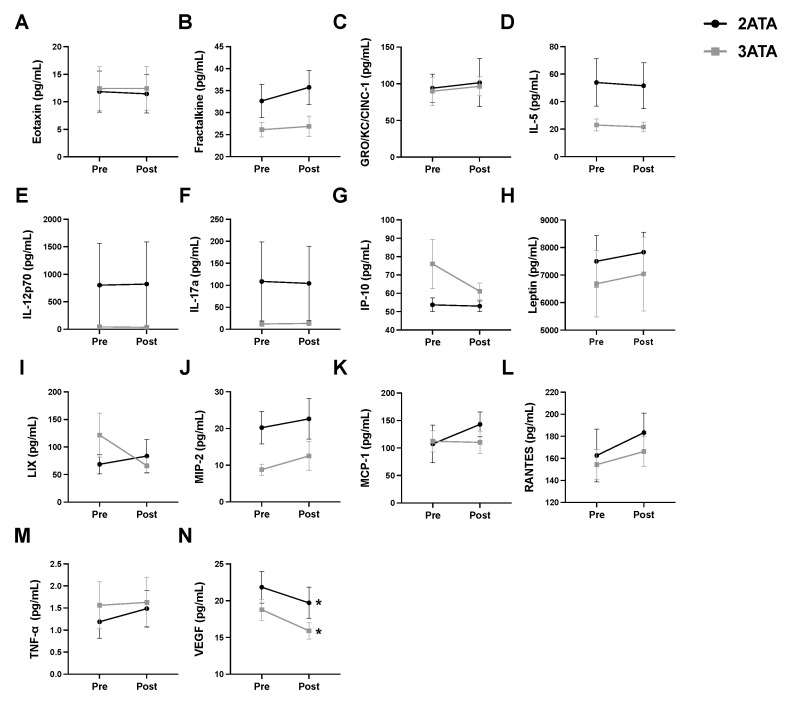
Alterations in Circulating Cytokines/Chemokines Pre- and Post-1-h of HBO Exposure at 2 or 3 ATA. Plasma levels of (**A**) eotaxin; (**B**) fractalkine; (**C**) GRO/KC/CINC-1; (**D**) interleukin-5 (IL-5); (**E**) interleukin-12p70 (IL-12p70); (**F**) interleukin-17a (IL-17a); (**G**) interferon gamma-induced protein 10 (IP-10 or CXCL10); (**H**) leptin; (**I**) LIX or C-X-C motif chemokine 5 (CXCL5); (**J**) macrophage inflammatory protein-2 (MIP-2); (**K**) monocyte chemoattractant protein-1 (MCP-1/CCL2); (**L**) regulated upon activation, normal T cell expressed and presumably secreted (RANTES); (**M**) tumor necrosis factor α (TNF-α) and (**N**) vascular endothelial growth factor (VEGF). Values are represented as means ± SEM. * = Post-HBO significantly different from Pre-HBO (*p* < 0.05).

To determine whether differences exist in the acute inflammatory responses to HBO pressures of 2 ATA and 3 ATA, changes in circulating levels of cytokines and chemokines were assessed immediately before and after a single 1-h exposure to HBO (Figure 1). Of the circulating proteins measured in the plasma, only VEGF was significantly altered, with post-HBO plasma levels significantly reduced compared to pre-HBO levels in both the 2 ATA and 3 ATA exposure groups.

### 3.2. Body Weight and Heart Weight Are Decreased with DOX Treatment

**Figure 2:**
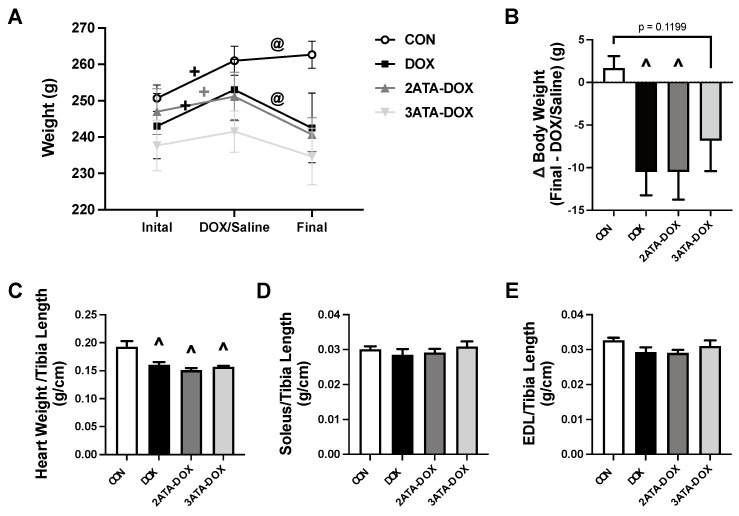
Body and Muscle Weights. Measurements of (**A**) change in group weight over the experimental timeline; (**B**) change in body weight (Final-DOX/Saline); (**C**) heart weight; (**D**) soleus weight; and (**E**) EDL weight. Muscle weights were all normalized to tibia length. Values are represented as means ± SEM. + = significantly different vs. Initial (*p* < 0.05). @ = significantly different vs. DOX/Saline (*p* < 0.05). ^ = significantly different vs. CON (*p* < 0.05).

Within group comparison of body weight over time showed an increase in mass in the CON, DOX and 2ATA-DOX groups when their initial (pre-HBO or room air exposure) weight was compared to their weight on the day of DOX/Saline treatment. Final body weight compared to DOX/Saline treatment was reduced in the DOX group only. (Figure 2A). When compared between groups, body weight change (Final-DOX/Saline) was greater in the DOX and 2ATA-DOX rats compared to CON (Figure 2B). Heart weight (normalized to tibia length) was significantly reduced in all groups treated with DOX compared to the saline treated CON group (Figure 2C). Soleus and EDL muscle weights were maintained between all groups (Figure 2D,E).

### 3.3. Cardiac Function Is Reduced with DOX and Not Improved by HBO Preconditioning

**Figure 3:**
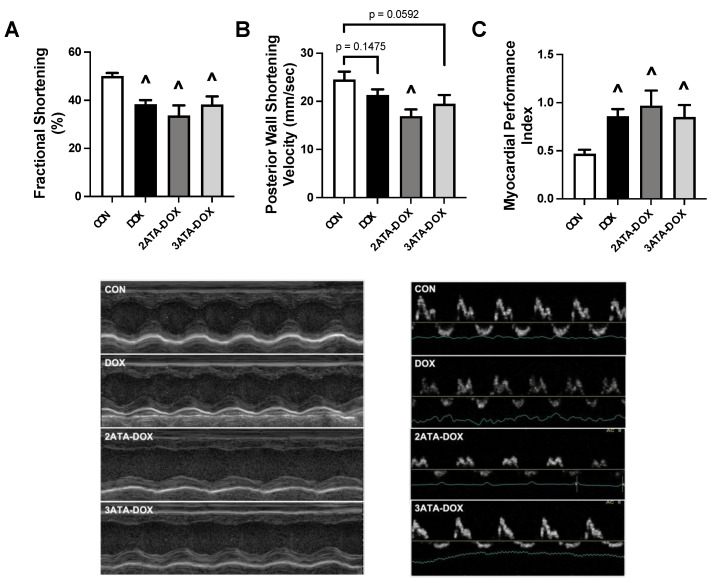
Cardiac Function. Measurement of (**A**) fractional shortening percentage; (**B**) posterior wall shortening velocity (PWSV); and (**C**) myocardial performance index (MPI). Representative images of M-mode (**left**) and Doppler (**right**) are shown below the graphs. Values are represented as means ± SEM. ^ = significantly different vs. CON (*p* < 0.05).

Echocardiography revealed significant systolic and diastolic myocardial dysfunction in all groups treated with DOX. LV fractional shortening percentage was reduced in all DOX-treated groups compared to CON (Figure 3A). PWSV was significantly reduced in the 2ATA-DOX group compared to CON, with trends for a reduction also apparent in the DOX (*p* = 0.1475) and 3ATA-DOX (*p* = 0.0592) groups compared to CON (Figure 3B). Assessment of the MPI revealed a significant increase in all DOX treated groups above CON (Figure 3C). The thickness of the posterior wall during systole and diastole (PWTs and PWTd) did not differ between groups. Septal wall thickness was significantly decreased in the 2ATA-DOX and 3ATA-DOX groups during systole (SWTs) compared to CON and 3ATA-DOX group compared to CON during diastole (SWTd) (Table 1).

### 3.4. Diaphragm Dysfunction Is Observed Following DOX Treatment

**Figure 4:**
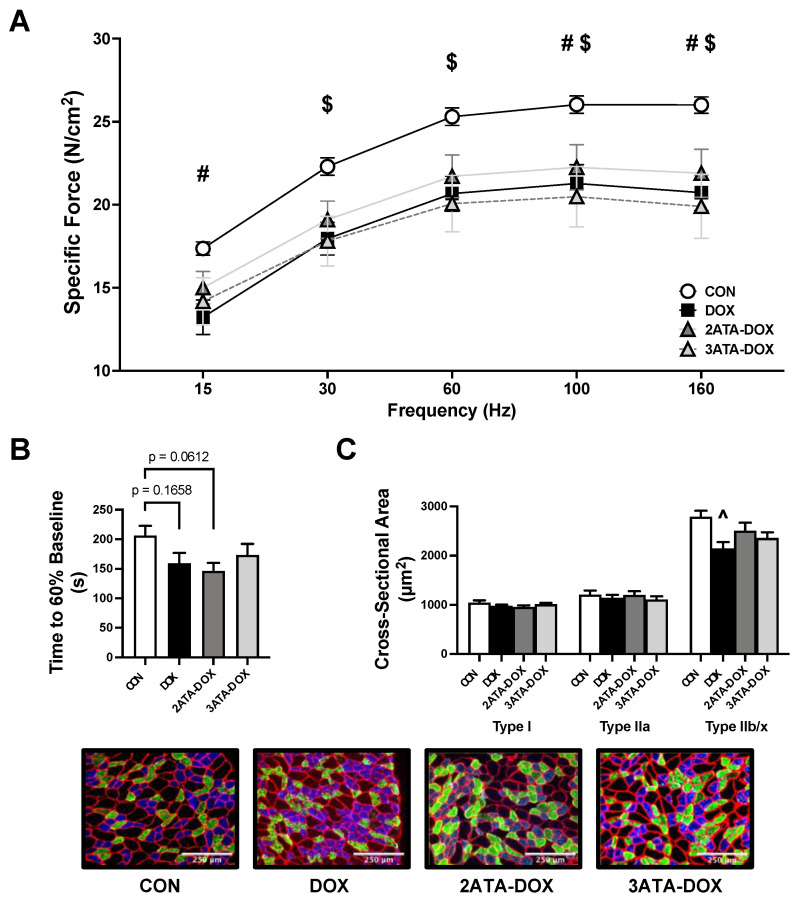
Diaphragm Function. Diaphragm measurement of (**A**) force-frequency response; (**B**) time to 60% of baseline force (fatigue); and (**C**) cross-sectional area of type I (blue), type IIa (green), and type IIb/x (black) fibers (representative images below). Values are represented as means ± SEM. # = CON significantly different vs. DOX (*p* < 0.05). $ = CON significantly different vs. 3ATA-DOX (*p* < 0.05). ^ = significantly different vs. CON (*p* < 0.05).

DOX treatment results in diaphragm weakness and contractile dysfunction, leading to respiratory complications, dyspnea and fatigue [37]. Our findings support this, as diaphragm force production in the DOX group was significantly reduced compared to CON at 15, 100 and 160 Hz (Figure 4A). Diaphragm specific force production was also significantly reduced in the 3ATA-DOX rats at frequencies between 30–160 Hz. However, there was no significant difference between the 2ATA-DOX group compared to CON. Time to 60% baseline was not significantly reduced in any of the DOX treated groups compared to CON (Figure 4B). Finally, DOX resulted in a significant reduction in the CSA of type IIb/x diaphragm muscle fibers compared to CON (Figure 4C). No differences existed in muscle fiber size in the 2ATA-DOX or 3ATA-DOX groups compared to CON.

### 3.5. DOX Promotes Mitochondrial Dysfunction and Reactive Oxygen Species Production

**Figure 5:**
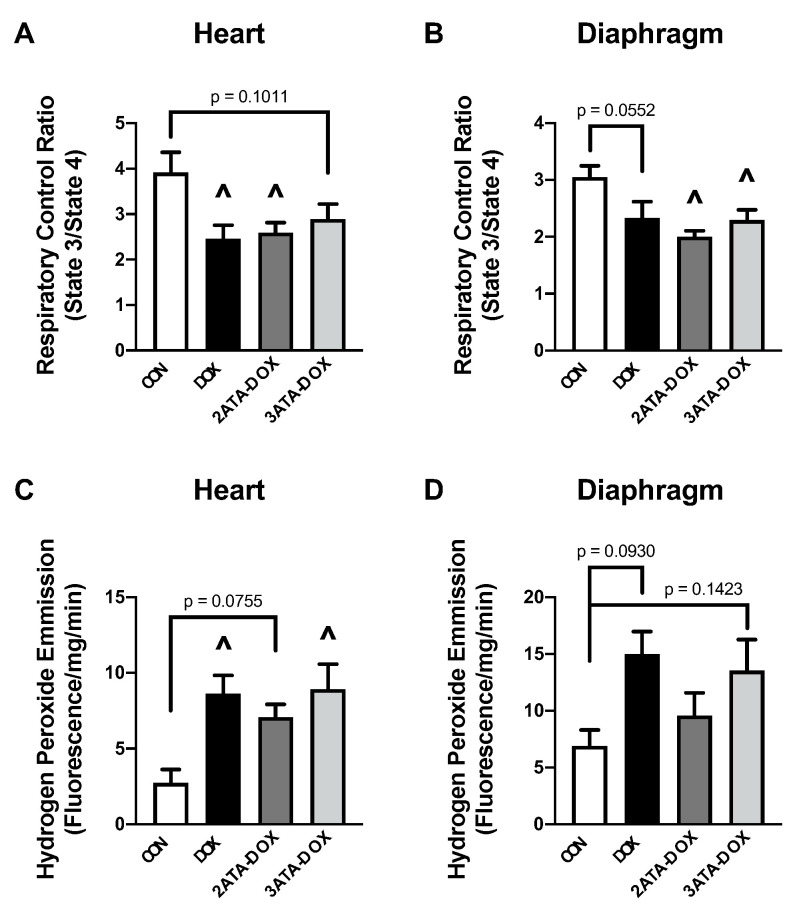
Mitochondrial Function and Oxidative Stress. Measurement of (**A**,**B**) mitochondrial respiratory control ratio and (**C**,**D**) mitochondrial hydrogen peroxide emission in permeabilized heart and diaphragm muscle fibers. Values are represented as means ± SEM. ^ significantly different vs. CON (*p* < 0.05).

Elevated ROS formation during HBO has been shown in animal and human studies, with evidence of both protective and harmful effects [38,39]. Regarding mitochondrial function, our results show a significant decrease in RCR in the DOX and 3ATA-DOX groups compared to CON in the heart (Figure 5A). Diaphragm mitochondrial function was significantly reduced in the 2ATA-DOX and 3ATA-DOX groups compared to CON (Figure 5B). Mitochondrial ROS production was increased in the DOX and 3ATA-DOX groups compared to CON in the heart (Figure 5C), while ROS emission did not reach significance between the CON and DOX treatment groups in the diaphragm (Figure 5D).

### 3.6. HBO Preconditioning Increases Muscle Antioxidant Capacity

**Figure 6:**
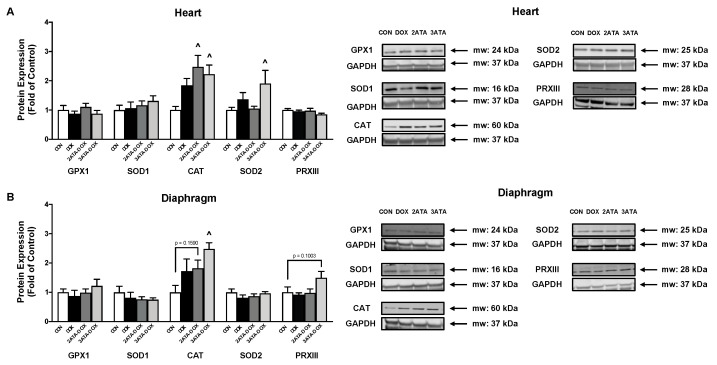
Antioxidant Expression. Protein expression of the endogenous antioxidants glutathione peroxidase 1 (GPX1), superoxide dismutase 1 (SOD1), catalase (CAT), superoxide dismutase 2 (SOD2), and peroxiredoxin III (PRXIII) in the (**A**) heart and (**B**) diaphragm. Representative Western blot images are shown to the right of the graphs. Values are represented as means ± SEM. ^ = significantly different vs. CON (*p* < 0.05).

HBO has been shown to increase muscle antioxidant expression [5,6]. Our results support these previous findings as 5 days of HBO preconditioning at 2 ATA and 3 ATA significantly increased catalase expression in the heart (Figure 6A). Exposure to 3 ATA HBO also resulted in increased SOD2 expression in the heart and increased catalase expression in the diaphragm (Figure 6B).

### 3.7. Alterations in Circulating Chemokines/Cytokines

**Figure 7:**
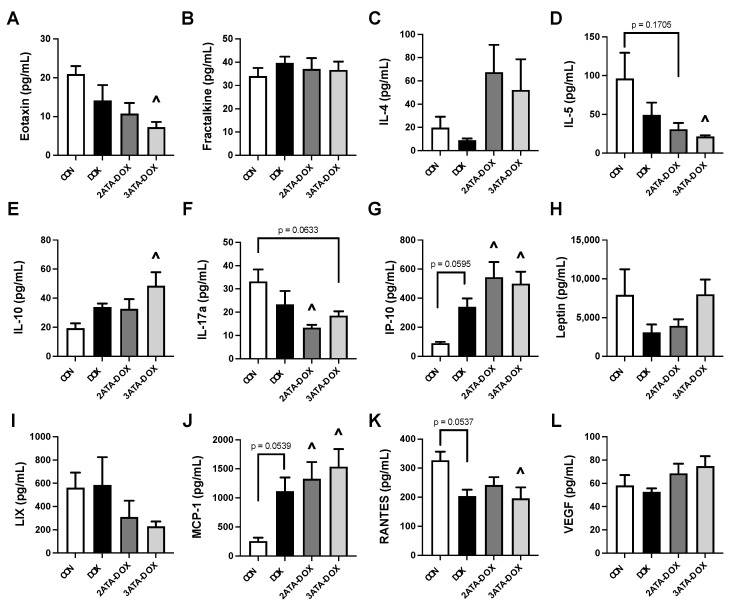
Circulating Cytokines/Chemokines. Plasma levels of (**A**) eotaxin; (**B**) fractalkine; (**C**) interleukin-4 (IL-4); (**D**) interleukin-5 (IL-5); (**E**) interleukin-10 (IL-10); (**F**) interleukin-17a (IL-17a); (**G**) interferon gamma-induced protein 10 (IP-10 or CXCL10); (**H**) leptin; (**I**) C-X-C motif chemokine 5 (CXCL5 or LIX); (**J**) monocyte chemoattractant protein-1 (MCP-1/CCL2); (**K**) regulated upon activation, normal T cell expressed and presumably secreted (RANTES); and (**L**) vascular endothelial growth factor (VEGF). (*p* < 0.05). Values are represented as means ± SEM. ^ = significantly different vs. CON (*p* < 0.05).

Analysis of plasma cytokines and chemokines showed altered levels primarily resulting from the combination of HBO preconditioning and DOX. DOX treatment alone did not significantly alter circulating expression of the cytokine/chemokine targets after 48 h (Figure 7). 2ATA-DOX induced an increase in the levels of the inflammatory proteins IP-10 and MCP-1 and a reduction in IL-17a. 3ATA-DOX elicited a greater response compared to CON, with increased levels of IP-10, MCP-1 and IL-10 and reduced levels of eotaxin, IL-5 and RANTES found in this group.

## 4. Discussion

Increasing evidence supports the use of HBO as an effective therapy to maintain muscle redox balance, prevent mitochondrial dysfunction and preserve muscle function [10,40]. While limited information exists regarding the effects of HBO treatment in combination with DOX chemotherapy, preclinical investigation reveals potential benefits to tumor growth reduction [41] and accelerated healing of extravasation injury [26]. A recent study also showed that HBO exposure prior to neoadjuvant chemotherapy with DOX did not adversely affect 5-year survival of patients with locally advanced breast cancer [42]. This study investigated the effects of HBO preconditioning on cardiac and respiratory muscle function following DOX administration. Our findings support the position that exposure to HBO therapy prior to DOX treatment is well-tolerated and does not exacerbate cardiorespiratory toxicity.

### 4.1. Inflammation, Oxidative Stress and Mitochondrial Function

HBO elevates ROS formation resulting in a modified inflammatory response, oxidative stress tolerance and mitochondrial oxidative phosphorylation [43,44]. Reported changes to the inflammatory process following HBO include both inhibition of pro-inflammatory cytokine production and accelerated immune response time [43,45,46]. Evaluation of plasma cytokines in our study provides additional evidence of the anti-inflammatory effects of HBO as exposure to a single 1-h HBO session at either 2 or 3 ATA showed a significant decrease in the level of pro-inflammatory cytokine VEGF in the plasma (Figure 1). Additionally, repeated HBO exposure for 5 days in DOX-treated rats resulted in a significant reduction in plasma levels of pro-inflammatory cytokines eotaxin, IL-5 and RANTES and increased levels of the anti-inflammatory protein IL-10 in rats exposed to HBO at 3 ATA (Figure 7).

Increased ROS production following HBO triggers activation of the oxidative stress-sensitive transcription factor Nrf2 to upregulate antioxidant enzyme gene expression and preserve redox homeostasis [47,48]. These antioxidant genes include SOD1, SOD2, catalase and GPX1, all of which are established targets of Nrf2 and are demonstrated to increase following HBO therapy [5,6,9,40,49]. Our data also show an HBO-specific antioxidant response with catalase protein expression increased in the diaphragm muscle and both SOD2 and catalase increased in the heart (Figure 6). These antioxidants play an important role in maintaining mitochondrial function. This is significant because the primary source of ROS in the heart and diaphragm muscles are the mitochondria and excessive oxidative stress significantly compromises oxidative phosphorylation and ATP production. In this regard, HBO therapy has been shown to improve electron transport chain complex activity, reduce membrane permeability and maintain ATP production under pathological conditions [50,51,52]. However, reports suggest that these beneficial effects may only occur when the therapy is given long-term [44]. It is hypothesized that when HBO therapy is limited to 5 or fewer sessions, cells reduce their mitochondrial activity to lower ROS production and reduce oxidative damage [53]. This finding supports the need for ongoing work to determine the appropriate dose of HBO needed for therapeutic benefit and may be associated with our findings that 5 days of HBO preconditioning did not improve mitochondrial function or ROS emission.

### 4.2. HBO Preconditioning and Cardiorespiratory Muscle Function

Exposure to repeated sessions of HBO therapy has the potential to protect the cardiorespiratory system with an increasing number of reports showing preservation of cardiac and diaphragm muscle function when used as a therapeutic countermeasure [5,17,40]. However, the use of HBO in combination with DOX is controversial stemming from seminal work by Upton et al. where 7 of 8 rats that were treated twice daily with HBO at 2 ATA died by the fourth week after DOX injection [24]. While the mortality rate in these rats was attributed to excessive free radical formation, this drastic effect on animal survival has not been replicated in subsequent studies [25,27]. Preliminary observations regarding the effects of HBO therapy concurrent with DOX treatment on cardiac function were published in 2008 when Karagoz et al. reported partial improvements in ejection fraction and fractional shortening in rats that had received 15 doses of DOX (1 mg/kg/dose) and a 90-min session of HBO at 2.4 ATA 5 days per week over a 4-week period [23]. Recently, a subsequent study showed elevated ROS production but preservation of cardiac structures with HBO preconditioning given daily for one week for 90 min at 2.5 ATA prior to DOX treatment [22]. Results from our study confirm these previous findings, as 5 days of HBO preconditioning at 2 or 3 ATA did not potentiate DOX-induced systolic or diastolic dysfunction (Figure 3). In addition, this study provides the first evidence that DOX-induced respiratory muscle dysfunction is not exacerbated by repeated HBO sessions (Figure 4). While HBO exposure did increase heart and diaphragm antioxidant capacity, these effects were not sufficient to reduce mitochondrial ROS production or preserve mitochondrial function. This finding could potentially be related to the short nature of the preconditioning protocol and the potential need for long-term HBO exposure to elicit the necessary mitochondrial adaptations to improve muscle contractile function following DOX exposure.

## 5. Conclusions

Although the clinical standpoint remains that HBO given in combination with DOX treatment should be avoided, and a minimum of a three day wash out recommended before starting HBO treatment [1], this study and other recent investigations support the concept that HBO does not potentiate DOX-induced cardiorespiratory muscle toxicity [22,23]. HBO may be beneficial not only for development as a therapeutic to reduce these negative consequences of DOX chemotherapy exposure, but there is also increasing evidence indicating that HBO therapy may be an effective adjuvant therapy to sensitize tumor cells to DOX [41]. Therefore, further work is needed to elucidate the optimal HBO therapy dosing protocol to maximize cardiorespiratory benefits when combined with DOX treatment.

## Figures and Tables

**Table 1:** Cardiac Function.

	CON	DOX	2ATA-DOX	3ATA-DOX
Heart Rate (bpm)	330.7 ± 13.8	347.2 ± 9.4	323.1 ± 7.7	344.3 ± 9.8
SWTd (mm)	1.79 ± 0.26	1.74 ± 0.11	1.61 ± 0.09	1.75 ± 0.10
SWTs (mm)	2.59 ± 0.27	2.57 ± 0.17	2.09 ± 0.02	2.40 ± 0.07
PWTd (mm)	1.72 ± 0.04	1.61 ± 0.05	1.60 ± 0.08	1.46 ± 0.07 ^
PWTs (mm)	2.76 ± 0.07	2.40 ± 0.08	2.32 ± 0.16 ^	2.17 ± 0.11 ^

Heart rate (beat per minute (bpm), septal wall thickness during diastole (SWTd), septal wall thickness during systole (SWTs), posterior wall thickness during diastole (PWTd) and posterior wall thickness during systole (PWTs)**.** Values are represented as means ± SEM. ^ = significantly different vs. CON (*p* < 0.05).

## Data Availability

Data is contained within the article.

## References

[1] Baude J., Cooper J.S. (2021). Hyperbaric contraindicated chemotherapeutic agents. StatPearls.

[2] Moon R.E. (2014). Hyperbaric oxygen treatment for decompression sickness. Undersea Hyperb. Med..

[3] Bhutani S., Vishwanath G. (2012). Hyperbaric oxygen and wound healing. Indian J. Plast. Surg..

[4] Thom S.R. (1989). Analytic Reviews: Hyperbaric Oxygen Therapy. J. Intensiv. Care Med..

[5] Smuder A.J., Turner S.M., Schuster C.M., Morton A.B., Hinkley J.M., Fuller D.D. (2020). Hyperbaric Oxygen Treatment Following Mid-Cervical Spinal Cord Injury Preserves Diaphragm Muscle Function. Int. J. Mol. Sci..

[6] Gregorevic P., Lynch G.S., Williams D.A. (2001). Hyperbaric oxygen modulates antioxidant enzyme activity in rat skeletal muscles. Eur. J. Appl. Physiol..

[7] Gregorevic P., Williams D.A., Lynch G.S. (2002). Hyperbaric oxygen increases the contractile function of regenerating rat slow muscles. Med. Sci. Sports Exerc..

[8] Gregorevic P., Lynch G.S., Williams D.A. (2000). Hyperbaric oxygen improves contractile function of regenerating rat skeletal muscle after myotoxic injury. J. Appl. Physiol..

[9] Oyaizu T., Enomoto M., Yamamoto N., Tsuji K., Horie M., Muneta T., Sekiya I., Okawa A., Yagishita K. (2018). Hyperbaric oxygen reduces inflammation, oxygenates injured muscle, and regenerates skeletal muscle via macrophage and satellite cell activation. Sci. Rep..

[10] Hadanny A., Hachmo Y., Rozali D., Catalogna M., Yaakobi E., Sova M., Gattegno H., Abu Hamed R., Lang E., Polak N. (2022). Effects of Hyperbaric Oxygen Therapy on Mitochondrial Respiration and Physical Performance in Middle-Aged Athletes: A Blinded, Randomized Controlled Trial. Sports Med. Open.

[11] Smuder A.J. (2019). Exercise stimulates beneficial adaptations to diminish doxorubicin-induced cellular toxicity. Am. J. Physiol. Regul. Integr. Comp. Physiol..

[12] Montalvo R.N., Doerr V., Nguyen B.L., Kelley R.C., Smuder A.J. (2021). Consideration of Sex as a Biological Variable in the Development of Doxorubicin Myotoxicity and the Efficacy of Exercise as a Therapeutic Intervention. Antioxidants.

[13] Wallace K.B., Sardao V.A., Oliveira P.J. (2020). Mitochondrial Determinants of Doxorubicin-Induced Cardiomyopathy. Circ. Res..

[14] Wallace K.B. (2003). Doxorubicin-induced cardiac mitochondrionopathy. Pharmacol. Toxicol..

[15] Leitman M., Efrati S., Fuchs S., Hadanny A., Vered Z. (2020). The effect of hyperbaric oxygenation therapy on myocardial function. Int. J. Cardiovasc. Imaging.

[16] Wunderlich T., Frey N., Kahler W., Lutz M., Radermacher P., Klapa S., Koch I., Tillmans F., Witte J., Koch A. (2017). Influence of hyperoxia on diastolic myocardial and arterial endothelial function. Undersea Hyperb. Med..

[17] Yogaratnam J.Z., Laden G., Guvendik L., Cowen M., Cale A., Griffin S. (2010). Hyperbaric oxygen preconditioning improves myocardial function, reduces length of intensive care stay, and limits complications post coronary artery bypass graft surgery. Cardiovasc. Revascularization Med..

[18] Nie H., Xiong L., Lao N., Chen S., Xu N., Zhu Z. (2006). Hyperbaric oxygen preconditioning induces tolerance against spinal cord ischemia by upregulation of antioxidant enzymes in rabbits. J. Cereb. Blood Flow Metab..

[19] Kim C.H., Choi H., Chun Y.S., Kim G.T., Park J.W., Kim M.S. (2001). Hyperbaric oxygenation pretreatment induces catalase and reduces infarct size in ischemic rat myocardium. Pflug. Arch..

[20] Yu S.Y., Chiu J.H., Yang S.D., Yu H.Y., Hsieh C.C., Chen P.J., Lui W.Y., Wu C.W. (2005). Preconditioned hyperbaric oxygenation protects the liver against ischemia-reperfusion injury in rats. J. Surg. Res..

[21] Cabigas B.P., Su J., Hutchins W., Shi Y., Schaefer R.B., Recinos R.F., Nilakantan V., Kindwall E., Niezgoda J.A., Baker J.E. (2006). Hyperoxic and hyperbaric-induced cardioprotection: Role of nitric oxide synthase 3. Cardiovasc. Res..

[22] Tezcan O., Karahan O., Alan M., Ekinci C., Yavuz C., Demirtas S., Ekinci A., Caliskan A. (2017). Hyperbaric Oxygen Preconditioning Provides Preliminary Protection Against Doxorubicin Cardiotoxicity. Acta Cardiol. Sin..

[23] Karagoz B., Suleymanoglu S., Uzun G., Bilgi O., Aydinoz S., Haholu A., Turken O., Onem Y., Kandemir E.G. (2008). Hyperbaric oxygen therapy does not potentiate doxorubicin-induced cardiotoxicity in rats. Basic Clin. Pharmacol. Toxicol..

[24] Upton P.G., Yamaguchi K.T., Myers S., Kidwell T.P., Anderson R.J. (1986). Effects of antioxidants and hyperbaric oxygen in ameliorating experimental doxorubicin skin toxicity in the rat. Cancer Treat. Rep..

[25] Firat O., Kirdok O., Makay O., Caliskan C., Yilmaz F., Ilgezdi S., Karabulut B., Coker A., Zeytunlu M. (2009). Can hyperbaric oxygenation decrease doxorubicin hepatotoxicity and improve regeneration in the injured liver?. J. Hepato-Biliary-Pancreat. Surg..

[26] Aktas S., Toklu A.S., Olgac V. (2000). Hyperbaric oxygen therapy in adriamycin extravasation: An experimental animal study. Ann. Plast. Surg..

[27] Monstrey S.J., Mullick P., Narayanan K., Ramasastry S.S. (1997). Hyperbaric oxygen therapy and free radical production: An experimental study in doxorubicin (Adriamycin) extravasation injuries. Ann. Plast. Surg..

[28] Thigpen J.T., Brady M.F., Homesley H.D., Malfetano J., DuBeshter B., Burger R.A., Liao S. (2004). Phase III trial of doxorubicin with or without cisplatin in advanced endometrial carcinoma: A gynecologic oncology group study. J. Clin. Oncol..

[29] Cai F., Luis M.A.F., Lin X., Wang M., Cai L., Cen C., Biskup E. (2019). Anthracycline-induced cardiotoxicity in the chemotherapy treatment of breast cancer: Preventive strategies and treatment. Mol. Clin. Oncol..

[30] Tibbles P.M., Edelsberg J.S. (1996). Hyperbaric-oxygen therapy. N. Engl. J. Med..

[31] Montalvo R.N., Doerr V., Kwon O.S., Talbert E.E., Yoo J.K., Hwang M.H., Nguyen B.L., Christou D.D., Kavazis A.N., Smuder A.J. (2020). Protection against Doxorubicin-Induced Cardiac Dysfunction Is Not Maintained Following Prolonged Autophagy Inhibition. Int. J. Mol. Sci..

[32] Powers S.K., Shanely R.A., Coombes J.S., Koesterer T.J., McKenzie M., Van Gammeren D., Cicale M., Dodd S.L. (2002). Mechanical ventilation results in progressive contractile dysfunction in the diaphragm. J. Appl. Physiol..

[33] Reid M.B. (2008). Free radicals and muscle fatigue: Of ROS, canaries, and the IOC. Free Radic. Biol. Med..

[34] Smuder A.J., Sollanek K.J., Nelson W.B., Min K., Talbert E.E., Kavazis A.N., Hudson M.B., Sandri M., Szeto H.H., Powers S.K. (2018). Crosstalk between autophagy and oxidative stress regulates proteolysis in the diaphragm during mechanical ventilation. Free Radic. Biol. Med..

[35] Min K., Kwon O.S., Smuder A.J., Wiggs M.P., Sollanek K.J., Christou D.D., Yoo J.K., Hwang M.H., Szeto H.H., Kavazis A.N. (2015). Increased mitochondrial emission of reactive oxygen species and calpain activation are required for doxorubicin-induced cardiac and skeletal muscle myopathy. J. Physiol..

[36] Smuder A.J., Hudson M.B., Nelson W.B., Kavazis A.N., Powers S.K. (2012). Nuclear factor-kappaB signaling contributes to mechanical ventilation-induced diaphragm weakness*. Crit. Care Med..

[37] Gilliam L.A., Moylan J.S., Callahan L.A., Sumandea M.P., Reid M.B. (2011). Doxorubicin causes diaphragm weakness in murine models of cancer chemotherapy. Muscle Nerve.

[38] Efrati S., Gall N., Bergan J., Fishlev G., Bass A., Berman S., Hamad-Abu R., Feigenzon M., Weissgarten J. (2009). Hyperbaric oxygen, oxidative stress, NO bioavailability and ulcer oxygenation in diabetic patients. Undersea Hyperb. Med..

[39] Tsuneyama K., Chen Y.C., Fujimoto M., Sasaki Y., Suzuki W., Shimada T., Iizuka S., Nagata M., Aburada M., Chen S.Y. (2011). Advantages and disadvantages of hyperbaric oxygen treatment in mice with obesity hyperlipidemia and steatohepatitis. Sci. World J..

[40] Oliveira M.S., Tanaka L.Y., Antonio E.L., Brandizzi L.I., Serra A.J., Dos Santos L., Krieger J.E., Laurindo F.R.M., Tucci P.J.F. (2020). Hyperbaric oxygenation improves redox control and reduces mortality in the acute phase of myocardial infarction in a rat model. Mol. Med. Rep..

[41] Wu X., Zhu Y., Huang W., Li J., Zhang B., Li Z., Yang X. (2018). Hyperbaric Oxygen Potentiates Doxil Antitumor Efficacy by Promoting Tumor Penetration and Sensitizing Cancer Cells. Adv. Sci.

[42] Heys S.D., Smith I.C., Ross J.A., Gilbert F.J., Brooks J., Semple S., Miller I.D., Hutcheon A., Sarkar T., Eremin O. (2006). A pilot study with long term follow up of hyperbaric oxygen pretreatment in patients with locally advanced breast cancer undergoing neo-adjuvant chemotherapy. Undersea Hyperb. Med..

[43] Benko R., Miklos Z., Agoston V.A., Ihonvien K., Repas C., Csepanyi-Komi R., Kerek M., Beres N.J., Horvath E.M. (2019). Hyperbaric Oxygen Therapy Dampens Inflammatory Cytokine Production and Does Not Worsen the Cardiac Function and Oxidative State of Diabetic Rats. Antioxidants.

[44] Schottlender N., Gottfried I., Ashery U. (2021). Hyperbaric Oxygen Treatment: Effects on Mitochondrial Function and Oxidative Stress. Biomolecules.

[45] Hedetoft M., Garred P., Madsen M.B., Hyldegaard O. (2021). Hyperbaric oxygen treatment is associated with a decrease in cytokine levels in patients with necrotizing soft-tissue infection. Physiol. Rep..

[46] Weisz G., Lavy A., Adir Y., Melamed Y., Rubin D., Eidelman S., Pollack S. (1997). Modification of in vivo and in vitro TNF-alpha, IL-1, and IL-6 secretion by circulating monocytes during hyperbaric oxygen treatment in patients with perianal Crohn’s disease. J. Clin. Immunol..

[47] Dhamodharan U., Karan A., Sireesh D., Vaishnavi A., Somasundar A., Rajesh K., Ramkumar K.M. (2019). Tissue-specific role of Nrf2 in the treatment of diabetic foot ulcers during hyperbaric oxygen therapy. Free Radic. Biol. Med..

[48] Yin X., Wang X., Fan Z., Peng C., Ren Z., Huang L., Liu Z., Zhao K. (2015). Hyperbaric Oxygen Preconditioning Attenuates Myocardium Ischemia-Reperfusion Injury Through Upregulation of Heme Oxygenase 1 Expression: PI3K/Akt/Nrf2 Pathway Involved. J. Cardiovasc. Pharmacol. Ther..

[49] Chen C., Chen W., Li Y., Dong Y., Teng X., Nong Z., Pan X., Lv L., Gao Y., Wu G. (2017). Hyperbaric oxygen protects against myocardial reperfusion injury via the inhibition of inflammation and the modulation of autophagy. Oncotarget.

[50] Dave K.R., Prado R., Busto R., Raval A.P., Bradley W.G., Torbati D., Perez-Pinzon M.A. (2003). Hyperbaric oxygen therapy protects against mitochondrial dysfunction and delays onset of motor neuron disease in Wobbler mice. Neuroscience.

[51] Zhou Z., Daugherty W.P., Sun D., Levasseur J.E., Altememi N., Hamm R.J., Rockswold G.L., Bullock M.R. (2007). Protection of mitochondrial function and improvement in cognitive recovery in rats treated with hyperbaric oxygen following lateral fluid-percussion injury. J. Neurosurg..

[52] Palzur E., Zaaroor M., Vlodavsky E., Milman F., Soustiel J.F. (2008). Neuroprotective effect of hyperbaric oxygen therapy in brain injury is mediated by preservation of mitochondrial membrane properties. Brain Res..

[53] Tezgin D., Giardina C., Perdrizet G.A., Hightower L.E. (2020). The effect of hyperbaric oxygen on mitochondrial and glycolytic energy metabolism: The caloristasis concept. Cell Stress Chaperones.

